# Influence of Speech and Language Therapy on Quality of Life in People With Primary Progressive Aphasia: A Scoping Review

**DOI:** 10.1111/1460-6984.70129

**Published:** 2025-09-22

**Authors:** Mirjam Gauch, Bianca Spelter, Katharina Geschke, Anna‐Lena Köb, Oliver Tüscher, Isabel Heinrich, Sabine Corsten

**Affiliations:** ^1^ Department of Psychiatry and Psychotherapy University Medical Center of the Johannes Gutenberg University Mainz Germany; ^2^ Faculty of Engineering and Health, Health Campus Göttingen University of Applied Sciences and Arts Hildesheim/Holzminden/Göttingen Hildesheim Germany; ^3^ Department of Psychiatry, Psychotherapy and Psychosomatic Medicine University Medicine Halle (Saale) of the Martin Luther University Halle‐Wittenberg Germany; ^4^ Faculty of Healthcare and Nursing Catholic Catholic University of Applied Sciences Mainz Mainz Germany

**Keywords:** aphasia, dementia, primary progressive aphasia, quality of life, speech and language therapy

## Abstract

**Background:**

There is evidence that interventions provided by a speech and language therapist (SLT) can positively impact the quality of life (QoL) of people with primary progressive aphasia (PPA). Current reviews refer to functional approaches rather than categorising QoL‐enhancing therapies.

**Aims:**

This paper aims to provide an overview of the approaches provided by SLTs to enhance QoL in people with PPA, taking into account influencing factors and various measurement instruments.

**Methods:**

Given the lack of concepts and inconsistent measurement instruments in the area of QoL‐enhancing therapies, the methodology of a scoping review was selected. Studies were identified through a broad database search in five databases (Medline, ScienceDirect, Speechbite, Psyndex and Cochrane). The research was further conducted using Google Scholar and handsearching of reference lists. The 244 studies identified were subjected to a duplicate check as well as a title, abstract and full‐text screening. The inclusion criteria were the presence of at least one person with PPA in the study sample, the described implementation of an intervention by at least one SLT, and QoL as a defined and measured outcome criterion. The Mixed Methods Appraisal Tool (MMAT) was used to assess the quality of the studies.

**Main Contribution:**

Twelve studies met the inclusion criteria. The studies showed evidence of an improved or stable QoL of the participants over the period of the respective interventions. Of the included studies, seven interventions took place in individual settings, three in group settings, and one in a dyadic constellation. In one study a combination of individual and group settings was used. The therapeutic approaches mainly aimed at more than one level of the Framework for Outcome Measurement. Most frequently covered were activities and participation (*n* = 9) and body function and body structure level (*n* = 7). The assessments used varied widely. The American Speech‐Language‐Hearing Association Quality of Communication Life Scale was used most frequently (*n* = 3). Other assessments included the Aphasia Impact Questionnaire‐21 or the Stroke and Aphasia Quality of Life Scale‐39, as well as interviews or self‐administered rating scales. The quality of the studies was heterogeneous and ranged from 2 to 5 out of a maximum of 5 according to MMAT criteria.

**Conclusions:**

The included studies demonstrate the potential of speech and language therapy to improve QoL in people with PPA and provide insights into influencing factors (e.g., treatment dose or setting of therapy). There is a need for high‐quality controlled trials of QoL‐enhancing interventions and for standardised QoL assessments tailored to the needs of people with PPA.

**WHAT THIS PAPER ADDS:**

*What is already known on this subject?*

- We know that speech and language therapy is valued by people with primary progressive aphasia (PPA) and has potential effects on their quality of life (QoL).

*What this paper adds to existing knowledge?*

- This scoping review provides an overview of the approaches provided by speech and language therapists, considering influencing factors and measurement instruments.

*What are the potential or actual clinical implications of this work?*

- Our research indicates a need for high‐quality controlled trials of QoL‐enhancing interventions. Focusing more on QoL could lead to the person‐centred approach that experts are already calling for.

## 1 Background

Primary progressive aphasia (PPA) is a neurodegenerative syndrome that is clinically defined by a gradual decline in linguistic functions with additional cognitive and/or behavioural deficits over the course of the disease (Mesulam et al. 2021; Warren et al. 2013). PPA is rare, with an incidence of approximately 3:100 000 (Coyle‐Gilchrist et al. 2016; Magnin et al. 2016), but the number of referrals for speech and language therapy is increasing (Volkmer et al. 2019; Henry and Grasso 2018), which indicates its growing importance in the worldwide healthcare system. A consensus paper defines the classification into a nonfluent (nfvPPA), semantic (svPPA) and logopenic (lvPPA) variant (Gorno‐Tempini et al. 2011). People with nfvPPA show agrammatism in their expressive speech and/or effortful speech accompanied by inconsistent phonetic errors. For those with svPPA, deficits in naming and single word comprehension interfere with everyday communication. People with lvPPA exhibit word‐finding difficulties in spontaneous speech, additionally, the absence of grammar and comprehension impairment is nowadays seen as a core feature combined with auditory processing deficits (Mesulam et al. 2021; Jiang et al. 2023). Beyond the three variants, there are patients remaining unclassifiable because of a less‐frequent combination of symptoms (mixed PPA, Mesulam et al. 2021).

Although the symptoms of PPA resemble those of post‐stroke aphasia (Taylor et al. 2014), the cause of PPA is focal dementia due to frontotemporal lobar degeneration and Alzheimer's pathology (Mesulam et al. 2012; Volkmer, Rogalski, et al. 2020). As PPA is often associated with early‐onset dementia and can affect people who are still working, family and financial problems may differ from those experienced by people with late‐onset dementia (Morhardt et al. 2015). For example, difficulties in communication lead to social withdrawal and impaired performance of social roles (Lo et al. 2022). Exploratory studies further suggest that a diagnosis of PPA is associated with reduced self‐efficacy and maladaptive coping strategies (Cartwright 2013). In approximately 40% of people with PPA, depression occurs during the course of the disease (Ruggero et al. 2019), which represents a greater prevalence than in people with Alzheimer's dementia (Medina and Weintraub 2007). Expert opinions and self‐reports from people with PPA emphasise the importance of interventions to improve quality of life (QoL), especially in view of the psychological stress caused by the diagnosis (Mooney et al. 2018; Roberts et al. 2022; Douglas 2023).

According to the World Health Organisation (WHO 1997), QoL is defined as an ‘individual's perception of their position in life in the context of culture and value systems in which they live and in relation to their goals, expectations, standards and concerns’ (World Health Organization 1997, 551). Core elements of QoL are multidimensionality and subjectivity. Numerous experts have attempted to further conceptualise QoL. However, no clear consensus was found (Cruice 2010). In a three‐stage Delphi process, patients with acute or chronic, physical or mental illness, relatives, clinicians and researchers were able to agree on at least 25 domains of QoL (Pietersma et al. 2013). There are different assessments measuring the QoL in different target groups. For example, the Stroke and Aphasia Quality of Life Scale‐39 (SAQOL‐39, Hilari et al. 2009) was selected as the core outcome assessment for the target group of people with post‐stroke aphasia because it is a reliable instrument for measuring the impact of elevated stress levels, diminished participation and impaired communication (Hilari, Wiggins, et al. 2003; Wallace et al. 2023).

The association between QoL and communication disorders has been discussed many times (Strong and Shadden 2020). While aphasia frequently affects QoL, neither the severity of language impairment nor the degree of improvement in language skills correlates with QoL (Franzén‐Dahlin et al. 2010). Therefore, in the case of aphasia, treatments specifically focusing on QoL have been developed as part of speech and language therapy. In the treatment of people with PPA, the focus on interventions to improve QoL is also particularly relevant given the negative prognosis of the degenerative disease (Mahendra and Tadokoro 2020).

The categorisation of therapeutic approaches for people with PPA is still inconsistent, and different terms are used (e.g., functional communication interventions vs. compensatory‐based approaches) (Volkmer, Rogalski, et al. 2020; Volkmer, Spector, et al. 2020). The Framework for Outcome Measurements (FROM, Simmons‐Mackie and Kagan 2007) from the Aphasia Institute might be suitable for orienting, as it distinguishes between (1) personal factors and identity, (2) body function and structure, (3) environment, and (4) activities and participation. These levels match with the levels of the International Classification of Functioning, Disability and Health (ICF), which is a framework introduced by the WHO in 2001 for measuring health and disability from a holistic perspective (World Health Organization 2013). Like the ICF, the FROM model could be used to allocate the focus of interventions in an internationally recognised way. At the centre of the FROM model and at the intersection of the different levels is QoL, the superior goal of aphasia therapy (Simmons‐Mackie and Kagan 2007). The FROM model has been used to conceptualise treatment of people with aphasia (Pitt et al. 2019) and has been applied in studies with people with PPA (Kim et al. 2018). Treatment pathways for people with PPA might cover several levels of the FROM model, because therapies need to be ‘sensitively tailored’ (Roberts et al. 2022, 1074) as the disease progresses, resulting in a more holistic approach (Volkmer, Rogalski, et al. 2020; Volkmer, Cartwright, et al. 2023).

Specific therapeutic approaches for people with PPA have been evaluated more intensively in recent years, focusing on the level of function and structure (Wauters et al. 2023). For example, it was shown that word retrieval therapy delivered by a speech and language therapist (SLT) can lead to significant and lasting functional improvement in early‐stage patients (Henry et al. 2019) or that therapeutic approaches targeting the discourse level can help patients to improve their informativeness and communication effectiveness (Kim 2017). However, three European surveys suggest that evidence‐based approaches are still needed to encourage SLTs to provide adequate services for people with PPA (Yaşa 2023; Volkmer et al. 2019; Battista et al. 2023).

A review by Ruggero et al. (2019) provided an overview of studies dealing with QoL in people with PPA. The authors identified an emerging research field with a lack of conceptualisation and described their work as a possible starting point for further research (Ruggero et al. 2019). The systematic review by Wauters et al. (2023) showed that the majority of intervention studies in people with PPA are still impairment‐based (e.g., Wauters et al. 2023; Battista et al. 2023). The authors excluded studies targeting psychological well‐being from their analysis and mention that research in this area has been small so far (Wauters et al. 2023). In another current systematic review, Watanabe et al. (2024) analysed therapeutic effects on QoL and communication function. The researchers focused on group interventions and excluded interventions provided in an individual setting (Watanabe et al. 2024).

Our scoping review aimed to provide an overview of speech and language therapy approaches enhancing QoL in people with PPA and to map the evidence of different methodologies. Various measurement instruments and factors influencing the effects of therapy were identified and investigated.

## 2 Methods

### 2.1 Study Design

This scoping review was conducted to describe current qualitative and quantitative research activities, to summarise research findings and to identify research gaps on QoL‐enhancing therapeutic approaches in speech and language therapy for people with PPA in preparation for our own future research (Gauch et al. 2024) regarding identity work for the target group.

### 2.2 Quality Framework and Reporting Standards

The scoping review is based on existing methodological guidance, including the following steps: definition of research targets and questions, development and adaptation of inclusion criteria, description of planned procedure, conduction of research, selection of evidence, data extraction, visualisation, summarising and reporting the results (Arksey and O'Malley 2005; Peters et al. 2021). The reporting of this scoping review followed the PRISMA Extension for Scoping Review checklist (ScR checklist, Supporting Information S1, Tricco et al. 2018). This scoping review does not have a registered protocol, a fact that is discussed in the limitations section.

### 2.3 Research Questions (RQ1‐3)

**RQ1**: Which approaches are available in speech and language therapy with people with PPA, focusing on QoL?
**RQ2**: How was the QoL of people with PPA measured in the studies?
**RQ3**: Which factors influencing the effects of therapy can be derived from the evidence?

### 2.4 Search Strategy

Five electronic databases (Medline, ScienceDirect, Speechbite, Psyndex and Cochrane) and Google Scholar were searched from inception until the 5 July 2023. Finally, reference lists of related reviews and included studies were screened for additional studies. The search was performed by the first author with the use of keywords and Medical Subject Headings (MeSH) terms where appropriate. No filters were used.

### 2.5 Search Terms

The main search terms were ‘primary progressive aphasia,’ ‘quality of life,’ ‘speech therapy,’ ‘therapy’ and ‘therapeutics.’ Further, the terms ‘health perception,’ ‘self‐esteem,’ and ‘sense of control’ were added as they mirror the distinct aspects of QoL which are especially relevant in the context of identity work. The term ‘satisfaction’ was added from the Delphi study by Pietersma et al. (2013) as it is a common outcome but often differentiated from QoL (Pietersma et al. 2013). Table 1 shows an example of the search strategy.

**TABLE 1:** Example of the search strategy.

	Search strings
#**1**	(“primary progressive aphasia”) AND (“quality of life” OR “satisfaction” OR “health perception” OR “self‐esteem” OR “sense of control”) AND (“therapy” OR “therapeutics” OR “speech therapy”)—No filters
#**2**	((“Aphasia, Primary Progressive”[Mesh]) AND “Quality of Life”[Majr]) AND “therapy” [Subheading]—No filters

### 2.6 The Eligibility Criteria

**FIGURE 1:**
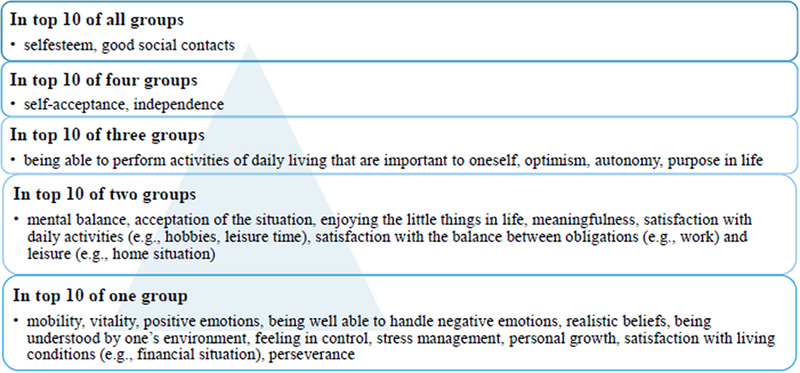
QoL domains according to Pietersma et al. (2013).

Quantitative and qualitative primary research papers were included if at least one person with PPA participated and if at least one trained SLT was responsible for the delivery of the intervention. Furthermore, the participation of people with other disorders or the participation of other professional groups did not constitute criteria for exclusion. The construct of QoL should be clearly named and measured. All studies covered at least one of the 25 domains agreed upon in the three‐stage Delphi consensus (Pietersma et al. 2013). The domains are listed in Figure 1 and were sorted according to the frequencies in the third round of the Delphi procedure.

Only studies published in English‐language journals were included. Articles were excluded if a full‐text version was not available or if the results of the studies remained unpublished (e.g., protocols).

### 2.7 Decision‐Making Regarding Article Inclusion

The studies identified by the search were subjected to a duplicate check as well as a title, abstract and full‐text screening by two independent screeners (first author and second author) against the pre‐determined inclusion criteria using the free web tool CADIMA (https://www.cadima.info/index.php). Any concerns about article inclusion were discussed until a consensus was reached.

### 2.8 Critical Appraisal

To assess the quality of included studies and to identify methodological gaps of current research, the Mixed Methods Appraisal Tool (MMAT, Hong et al. 2018) was completed by the two screeners. The MMAT contains two screening questions, which, if answered ‘No,’ may indicate that the evaluated study is not an empirical study and therefore cannot be assessed. Furthermore, the checklist contains five categories of study designs with five methodological quality criteria each (Crowe and Sheppard 2011). A unique feature of the MMAT is that it considers mixed methods research. According to the authors of the MMAT, mixed methods involve a minimum combination of one qualitative and one quantitative method. Both quantitative and qualitative methods should be carried out according to the criteria of the research tradition invoked, and the integration must be defined a priori and throughout all phases of the research process. Furthermore, all studies included were allocated to a level of evidence according to the National Health and Medical Research Council (NHMRC) hierarchy (Merlin et al. 2009). This hierarchy ranks studies according to their risk of bias and methodological strength by using Roman numerals. The designations are listed in Table 2.

**TABLE 2:** Designations of levels of evidence according to the National Health and Medical Research Council (Merlin et al. 2009).

Level of evidence	Study design
I	Evidence obtained from a systematic review of all relevant randomised controlled trials
II	Evidence obtained from at least one properly designed randomised controlled trial
III‐1	Evidence obtained from well‐designed pseudorandomised controlled trials
III‐2	Evidence obtained from comparative studies with concurrent controls and allocation not randomised, cohort studies, case‐control studies, or interrupted time series with a control group
III‐3	Evidence obtained from comparative studies with historical control, two or more single arm studies, or interrupted time series without a parallel control group
IV	Evidence obtained from case series, either post‐test or pretest/post‐test

In our study, the MMAT checklist was completed by two independent researchers (first and second authors) in an Excel 365 sheet (version 2204, Microsoft). Following the independent assessment, the ratings were compared, and a consensus was reached when the raters disagreed. The levels of evidence were determined by the same two independent researchers (first and second authors), and inconsistencies were likewise resolved via consensus.

### 2.9 Data Extraction

**FIGURE 2:**
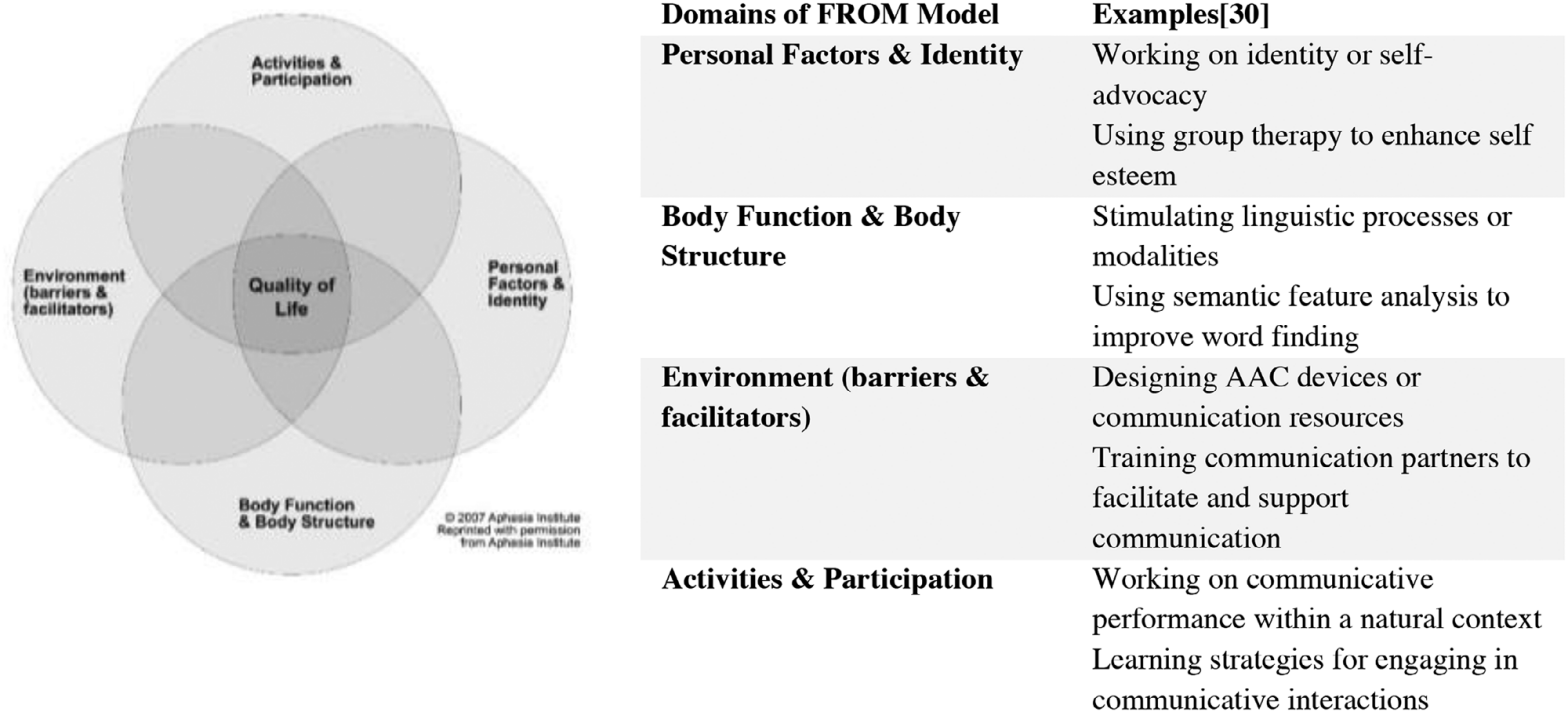
Framework for Outcome Measurement (FROM). With permission from the Aphasia Institute, Toronto, Ontario, Canada (AAC = augmentative and alternative communication).

To evaluate the included studies with the MMAT, a categorisation into types of study design was necessary. The following criteria for data extraction were developed a priori: ‘participants,’ ‘intervention,’ ‘dose,’ ‘setting,’ ‘professionals,’ ‘QoL‐related assessments’ and ‘results.’ The categories ‘data collection’ and ‘data analysis’ were added at a later stage. The category ‘participants’ included the number of people with PPA, a description of the variants (nfvPPA, svPPA, lvPPA or mixed PPA [Gorno‐Tempini et al. 2011]), relevant comorbidities such as depression, the sample size, and the inclusion of relatives. The category ‘intervention’ contained a description of procedures and materials used. To obtain a more comprehensive picture of the therapeutic approaches, the FROM model was used. The approaches were allocated to the four levels of the FROM model according to their primary orientation. Data extraction and allocation of research to the levels of the FROM model were conducted by the first author only. Examples of the different levels are given in Figure 2.

Further information about the setting (individual setting, group setting, dyadic setting, or a combination of different settings) of the included studies was extracted. The findings were described narratively.

## 3 Results

### 3.1 Article Inclusion

**FIGURE 3:**
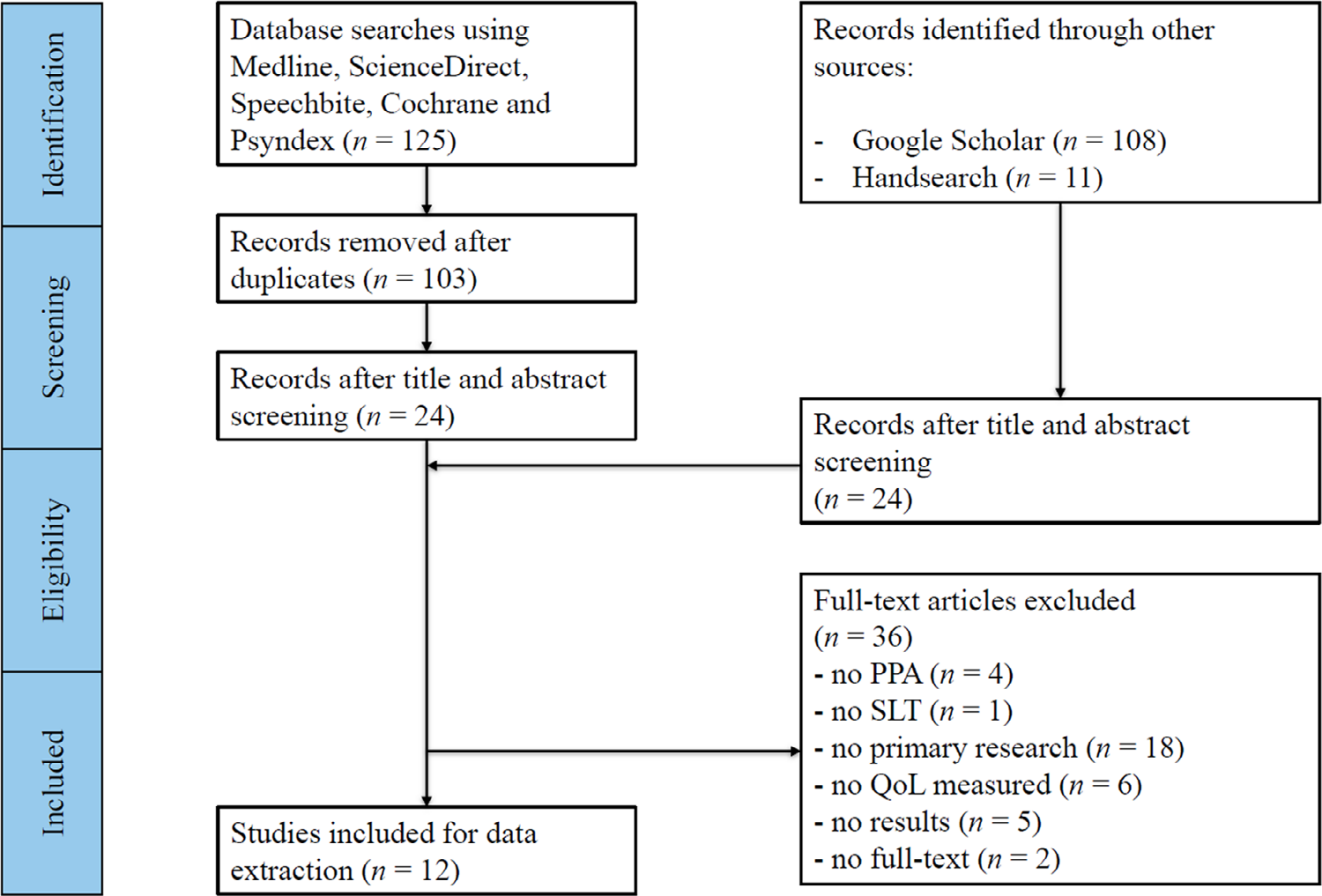
PRISMA flow chart (PPA = primary progressive aphasia, QoL = quality of life, SLT = speech and language therapist).

The literature search yielded a total of 244 papers. Records identified through database searches (*n* = 125) were checked for duplicates. After removing duplicates, 103 studies were screened according to title and abstract, resulting in 24 studies meeting the inclusion criteria. Further records were identified through Google Scholar (*n* = 108) and handsearching of reference lists (*n* = 11). Of these, another 24 studies were integrated into the full text screening. After reading the full texts of 48 articles, 36 articles were excluded as they did not fulfil eligibility criteria, resulting in 12 papers included for data extraction. See Figure 3, the PRISMA flow diagram, for an overview.

### 3.2 Description and Critical Appraisal of Included Studies

Study quality was heterogeneous and ranged from 2 out of a maximum of 5 MMAT criteria. The maximum possible MMAT criteria were reached by seven of the selected studies. The level of evidence ranged from II to IV according to the NHMRC hierarchy. The results of the MMAT assessment and the evidence levels assigned are shown in Table 3. An overview of the extracted data can be found in Table 4. At the end of the results section, the key results are summarised.

**TABLE 3:** Results of the critical appraisal.

No.	Author, year	Study design	Criteria met according to MMAT	Level of evidence
1	Kim et al. (2018)	Qualitative	5 out of 5	IV
2	Morhardt et al. (2019)	Qualitative	5 out of 5	IV
3	Jokel et al. (2017)	Quantitative, nonrandomised	3 out of 5	III‐2
			(2 unclear)	
4	Góral‐Półrola et al. (2015)	Quantitative descriptive	3 out of 5	IV
			(2 unclear)	
5	Rogalski et al. (2016)	Quantitative descriptive	5 out of 5	III‐3
6	Volkmer et al. (2023)	Quantitative, randomised‐controlled	5 out of 5	II
7	Jokel et al. (2010)	Quantitative descriptive	5 out of 5	IV
8	Whitworth et al. (2018)	Quantitative descriptive	5 out of 5	IV
9	Beales et al. (2016)	Quantitative descriptive	4 out of 5	IV
10	Mahendra and Tadokoro (2020)	Quantitative descriptive	2 out of 5	IV
			(3 unclear)	
11	Cadório et al. (2021)	Quantitative descriptive	5 out of 5	IV
12	Andrade‐Calderón (2015)	Quantitative descriptive	2 out of 5	IV
			(2 unclear)	

*Note*: A description of ‘unclear’ was assigned if information was not available from the study.

Abbreviation: MMAT, mixed methods appraisal tool.

**TABLE 4:** Summary of the included studies.

	Participants’							Data		
Author, year	sample	sex	age	Interventions	Dose	Setting	Professionals	collection	analysis	Results
Kim et al. (2018)	1x lvPPA (1x care partner)	1x female (1x male)	62 years. (68 years)	Aphasia Camp facilitates a variety of activities (e.g., adapted archery, canoeing, orienteering, hiking, caregiver support and conversation groups)	Once a year over a weekend	Group	Clinicians/students from the fields of speech and language therapy, physical therapy and occupational therapy	Exploratory semi‐structured interviewing	Conventional content analysis (inductive approach)	The couple reported an increase in social connections and the launch of new activities. The couple's personality traits had an impact on their experience of aphasia camp and how they integrated their experience into their everyday lives after the camp
Morhardt et al. (2019)	*Pilot phase*: 6x PPA *Formal intervention*: 9x PPA (8x care partner)	*Pilot phase*: 2x female, 4x male *Formal intervention*: 4x female, 5x male (6x female, 2x male)	*Pilot phase*: 53–80 years, *M* = 67.5 *Formal intervention*: 55–82 years, *M* = 67.2	*Pilot phase*: ‘PPA education/talk‐based support group (Yale 1999)’ (focus on counselling, patient education and introduction of AAC techniques) with a separate care partner group *Formal intervention*: ‘Hybrid Support Group’ for people with PPA and their care partners (focus similar to *Pilot phase*) ‘Social Activity Group’ for people with PPA (focus on activities including art or horticulture)	Five 90‐min bimonthly sessions	Group	SLT, clinical neuropsychologist, cognitive neuroscientist	Observational field notes	Thematic analysis	Support and education groups were valued by participants and contributed to a sense of wellbeing, personal empowerment and QoL (‘One member shared that he was "very depressed before the group" and "intermediately depressed after the group,"’ 1316).
Jokel et al. (2017)	2x lvPPA 3x nfvPPA (5x care partners)	2x female 3x male (3x female, 2x male)	59–80 years, *M* = 72.4 (56–85 years, *M* = 70.4)	*PPA participant group*: conversations about current global, local or personal events without focus on health issues; word‐retrieval strategies *Caregiver group*: networking, speaking about current needs of partners; 2 of 10 sessions are practice of communication strategies in spousal dyads	Ten 120‐min weekly sessions	Group	SLT, students from the field of speech and language therapy, social worker, neuropsychologist, nutrition scientist, neuropsychiatrist, neurorehabilitation scientist, clinical psychologist	Self‐assessment scale; spousal questionnaire; use of communication strategies; qualitative feedback	Inferential statistics (using the Wilcoxon sign‐ranked test); descriptive statistics	Most valuable aspects for people with PPA and their partners were: disease education, learning about therapy options/psychosocial aspects and interdisciplinarity. Discussing how to deal with PPA was important. Participants described the group as a ‘fantastic experience’ (60) and wished to continue the programme beyond the initial 10 weeks. Comparison of pre‐post intervention scores in ASHA QCLS suggests positive changes (*W* = 0, *Z* = ‐2.0226, *p* < 0.05, *r* = 0.64).
Góral‐Półrola et al. (2015)	1x nfvPPA	1x female	73 years	Training with an AAC system specially designed for the needs of the patient	20 sessions	Individual	AAC therapist/speech and language therapist, nursing staff	Documentation of conversational speech	Qualitative analysis, descriptive statistics	The patient had the ability to communicate nonverbally with her son and the nursing staff.
Rogalski et al. (2016)	28x PPA 3x other dementia diagnoses	18x female, 13x male	56–83 years *M* = 67.2	Internet‐based therapy via the Communication Bridge Web application, including impairment‐based/activity‐ and participation‐based approaches/disease education/counselling	Eight 60‐min sessions	Individual	SLT	Self‐reported functional gains, self‐assessment scale, qualitative interviews	Inferential statistics (using analysis of variance, *t*‐test), descriptive statistics	Participant, care partner, and therapist feedback were overwhelmingly positive, with 16 participant and/or care partner pairs reporting that therapy ‘exceeded’ expectations. Repeated‐measures ANOVA showed a significant change in CCRSA scores across the three test intervals (*p* = 0.02). Post hoc *t*‐tests revealed a significant improvement in their confidence in communication from baseline to the 2 months (*p* = 0.018).
Volkmer, Walton et al. (2023)	4x nfvPPA 3x lvPPA 2x mixed PPA (9x care partner)	5x female 4x male (5x female, 4x male)	57–85 years *M* = 72.1 (34–80, *M* = 64.6)	Communication partner training in which a dyad is supported to reflect on a video sample of their own conversation, identifying behaviours that facilitate or hinder communication; role plays and homework tasks enable transfer	6 weeks of intervention containing four 60‐min sessions	Dyads	Trained SLT	Self‐assessment scale; communication partner rating scale; 4 × 10‐min videos of recorded everyday conversation	Descriptive statistics (using minimally clinically important difference)	29/30 intervention goals achieved, and 16 of 30 coded conversation behaviours demonstrated change in the right direction; both (intervention and control) groups experienced an improvement in QoL.
Jokel et al. (2010)	1x svPPA	1x male	56 years^2^	Retraining of forgotten words through errorless learning; utilizing the MossTalk Words software	Three times a week, 60‐min sessions	Individual	SLT	Linguistic/cognitive testing; word lists; rating scale	Inferential statistics (using the McNemar and Wilcoxon sign‐ranked test)	The results of the Wilcoxon test indicate non‐significant in ASHA QCLS scores (*p* = 0.125).
Whitworth et al. (2018)	1x lvPPA 1x svPPA	1x female 1x male	54–59 years *M* = 56.5	NARNIA intervention programme (Whitworth et al. 2015)	20 sessions over a 10‐week period	Individual	SLT	Linguistic/cognitive testing; discourse performance using the *Curtin University Discourse Protocol*	Discourse analysis; inferential statistics (using McNemar, Fisher's exact test and the reliable change index)	Significant changes were seen in self‐reported social communication and participation ratings on the ALA according to the Reliable Change Index (RCI, ≥ 1.96 with a confidence interval of 95%).
Beales et al. (2016)	1x lvPPA 3x svPPA	1x female 3x male	53–70 years *M* = 60.25	Self‐cueing lexical retrieval intervention across word classes (nouns, verbs and adjectives)	Eight 90‐min therapy sessions twice weekly	Individual	SLT	Naming and discourse performance using the *Curtin University Discourse Protocol, s*elf‐assessment scale	Inferential statistics (using the Cochran test, McNemar test, and Fisher exact test), and descriptive statistics	All participants rated their confidence in word retrieval skills higher after intervention (range of 10% to 40% improvement) and perceived that their ability of word retrieval also improved.
Mahendra and Tadokoro (2020)	1x nfvPPA	1x female	57 years	Range of interventions offered to support communicative function, social participation, sense of identity, and emotional well‐being	Over a period of 3 years	Combined	SLT	Linguistic/cognitive testing, self‐assessment scale	Descriptive statistics	Improved QoL‐scores in ASHA QCLS between Years 1 and 2 (3/5 > 4/5). In Year 3, the participant reported feeling positive about the future, confident and having positive things to do but also feeling socially isolated and bored.
Cadório et al. (2021)	1x mixed PPA 1x nfvPPA	1x female 1x male	52–60 years *M* = 56	Structured training	12 sessions	Individual	SLT	Linguistic/cognitive testing, self‐assessment scale	Inferential statistics (using the McNemar test)	The patients’ QoL remained steady (C1 scored 18/25 at both time points; C2 scored 17/25 before and 18/25 after treatment).
Andrade‐Calderón (2015)	1x nfvPPA	1x male	84 years	Stimulating the phonological, lexical and syntactic processing	Weekly therapy sessions for a period of 12 months	Individual	SLT	Linguistic/cognitive testing, self‐assessment scale	Descriptive statistics	Continuous improvement in GDS‐scores throughout the intervention (12 > 8 > 5); improvements in NPI (1 > 0 > 0); ‘Good’ and stable QoL according to DEMQOL. Family members of the participant reported improved participation in social life and that the participant lost his fear of initiating a conversation with relatives or strangers.

Abbreviations: AAC, augmentative and alternative communication; ASHA QCLS, American Speech‐Language‐Hearing Association Quality of Communication Life Scale; DEMQOL, dementia quality of life measure; GDS, Geriatric Depression Scale; lvPPA, logopenic variant of PPA; NARNIA, novel approach to real‐life communication: narrative intervention in aphasia; nfvPPA, nonfluent variant of PPA; NPI, neuropsychiatric inventory; PPA, primary progressive aphasia; QoL, quality of life; SLT, speech and language therapist; svPPA, semantic variant of PPA.

#### 3.2.1 Qualitative Studies

Two of the included studies were categorised as qualitative and contained case studies (Kim et al. 2018; Morhardt et al. 2019). The ‘cases’ were one person with PPA (*n* = 1) in one study and one group (*n* = 9) in the other. In both studies, the intervention was conducted in a group setting (Kim et al. 2018; Morhardt et al. 2019). Outcomes were collected by interviews (Kim et al. 2018) and observational field notes using a semistructured format (Morhardt et al. 2019). The quality of the two qualitative studies was highly rated by the researchers with 5 out of 5 of the MMAT criteria. The level of evidence was IV. Both studies are described below.

Kim et al. (2018) showed the results of a semistructured interview conducted with a person with PPA and her partner. Both had taken part in an almost 3‐day aphasia camp, which was a programme mainly attended by people with post‐stroke aphasia. The interview guide was based on the adapted version of the FROM model. Data analysis revealed positive effects of the Aphasia Camp on the QoL, including both increased social contact and the need for new activities. It has also been suggested that couples’ personality traits (‘positivity, resilience, and proactiveness’) reinforce these effects (Kim et al. 2018, 278).

Morhardt et al. (2019) described the development, piloting and evaluation of a psychoeducational support programme for people with PPA and their care partners. The intervention consisted of five bimonthly 90‐min sessions. In the pilot phase, six people with PPA participated, and in the evaluation phase, nine people with PPA and eight relatives participated in the programme. The results consisted of the following topics: coping with limitations and language decline, expression of resilience, stigmatisation, experience of self‐confidence, and the development of a group feeling. In particular, the education session conducted by the SLT was appreciated by participants, as the ‘practical strategies provided a sense of hopefulness to people with PPA’ (Morhardt et al. (2019, 1324). Furthermore, the participants valued social and creative arts‐based activities or horticultural therapy. The authors concluded that their psychoeducational support programme was feasible and had positive effects on wellbeing and QoL (Morhardt et al. 2019).

#### 3.2.2 Quantitative Randomised Controlled Studies

One of the included studies was a quantitative randomised controlled study (Volkmer, Walton, et al. 2023). The quality was highly rated by the researchers with 5 out of 5 of the MMAT criteria. The level of evidence was II.

In this study, Volkmer, Walton et al. (2023) presented the results of their pilot study of a communication partner training programme for people living with PPA and their main conversation partner, typically a family member or friend (‘a dyad’). Eighteen people with PPA plus their communication partners were randomised into an intervention group or a control group. The dyads in the intervention group received four sessions of 60 min of communication partner training, and the participants in the control group received no SLT treatment. Local SLTs were trained to deliver the programme. The fidelity of a random sample of video recordings was assessed by two SLT students, whereby a high fidelity (87.2%) was determined. Multiple assessments, such as the Aphasia Impact Questionnaire‐21 (AIQ‐21), Dementia Quality of Life Measure (DEMQOL), and Communication Confidence Rating Scale for Aphasia (CCRSA), were used to measure the QoL of participants with PPA. The Perceived Stress Scale (PSS) and Zarit Burden Scale were used to assess the experience of the participants’ communication partners. The AIQ‐21 scores showed a positive trend with regard to the burden of disease in the intervention group only. The DEMQOL and CCRSA indicate improvements in QoL and communicative self‐confidence in the intervention and control groups (Volkmer, Walton, et al. 2023).

#### 3.2.3 Quantitative Nonrandomised Studies

One of the included studies was quantitative and nonrandomised and included qualitative comments from participants (Jokel et al. 2017). The quality was rated as average, with 3 out of 5 MMAT criteria met. Measurements were estimated as appropriate, outcome data were complete, and due to the comparability of the groups, confounding bias was evaluated as low. It is not clear if the selected participants are representative of the target population. Furthermore, possible modifications of the manual are not described. The level of evidence was III‐1.

In this study, Jokel et al. (2017) report the results of a structured group intervention for people with PPA and their partners. Ten participants with PPA and their partners were allocated on a first‐come‐first‐served basis to the treatment and control groups. The participants of the intervention group attended 10 weekly sessions lasting 120 min each; participants in the control group did not receive any treatment. In eight out of 10 sessions, people with PPA and their partners were treated in separate groups. Two sessions were held in a dyadic group setting. The interventions included language exercises, compensation strategy training, counselling, and education. The outcome measures were qualitative feedback and the American Speech‐Language‐Hearing Association Quality of Communication Life Scale (ASHA QCLS). Furthermore, a partner questionnaire and an examination of communication strategy use were conducted. The quantitative results indicate a significant improvement in the ASHA QCLS in the treatment group, and the qualitative feedback showed positive effects in the areas of increased knowledge, awareness of the progression of the disease and dealing with psychosocial difficulties (Jokel et al. 2017).

#### 3.2.4 Quantitative Descriptive Studies

Eight of the included studies were categorised as quantitative descriptive: seven case studies (Mahendra and Tadokoro 2020; Góral‐Półrola et al. 2015; Andrade‐Calderón et al. 2015; Beales et al. 2016; Cadório et al. 2021; Jokel et al. 2010; Whitworth et al. 2018) and one quantitative study without a control group (Rogalski et al. 2016). The sample sizes of the eight quantitative descriptive studies ranged from one individual (Mahendra and Tadokoro 2020) to 34 participants (Rogalski et al. 2016). Six out of seven case studies referred to a specific approach (e.g., self‐cueing lexical retrieval intervention), whereby Mahendra and Tadokoro (2020) showed the impact of a multi‐component and combined approach with individual and group therapies in the course of a person with nfvPPA over 3 years. The quality of the quantitative descriptive studies was evaluated as moderate to high. Rogalski et al. (2016) performed one quantitative study without a control group with 5 out of 5 MMAT criteria. The level of evidence was III‐3. Evaluation of the seven single case studies yielded from 2 to 5 out of 5 MMAT criteria. The level of evidence was IV. The eight studies are described below.

Góral‐Półrola et al. (2015) provided insight into the use of augmentative and alternative communication (AAC) originally designed for a non‐verbal person with nfvPPA. The AAC system was customised to meet individual needs to enable communication. After 20 sessions, the participant was able to use nonverbal communication independently. With the help of the Non‐verbal Communication Scale, a notable increase in non‐verbal communication was measured. The positive effect of the intervention on QoL was based on observations. The authors describe how the participant revitalised social contacts (Góral‐Półrola et al. 2015).

Rogalski et al. (2016) investigated the feasibility of internet‐based speech and language therapy for people with progressive aphasia (e.g., PPA). The study included 34 participants, three of whom dropped out during the course of the study. Participants received eight person‐centred therapy sessions in an individual setting via the Communication Bridge Web application. The online therapy sessions included impairment‐, activity‐, or participation‐based approaches as well as ongoing disease education and counselling. Additionally, participants received home exercises on their personalised Communication Bridge Homepage. QoL‐related assessments included the Communication Confidence Rating Scale for Aphasia (CCRSA), the American Speech‐Language‐Hearing Association functional communication measures (ASHA‐FCM) and a semistructured interview. Repeated‐measures ANOVA revealed a significant improvement in CCRSA scores across pre‐test, post‐test and follow‐up (*p* = 0.02). Post hoc *t*‐tests showed a significant improvement in confidence in communication from baseline to 2 months post‐enrolment. In a second analysis, Rogalski et al. (2022) utilised a mixed linear model to demonstrate that positive effects on communication confidence were only evident among participants with engaged communication partners (Rogalski et al. 2016).

Jokel et al. (2010) showed the feasibility and effectiveness of an errorless learning approach via computer‐based therapy in one participant with svPPA. The participant received three therapy sessions per week for 60 min each according to the MossTalk Words therapy programme. MossTalk Words is a software‐based treatment system containing 340 words with corresponding pictures and cues that enables structured word retrieval therapy (Fink et al. 2002). The QoL was assessed by the ASHA QCLS. There was no statistically significant change in QoL (*p* = 0.125) throughout the intervention (Jokel et al. 2010).

Whitworth et al. (2018) evaluated the effects of a discourse intervention on everyday speech for two individuals with PPA (svPPA and lvPPA). The participants received 20 sessions over a 10‐week period according to the novel approach to real‐life communication: Narrative Intervention in Aphasia (NARNIA, Whitworth et al. 2015). NARNIA targets speech production at the word, sentence and discourse levels and therefore facilitates the transfer of functional gains into everyday speech. The Reliable Change Index^1^ (RCI, ≥ 1.96) was identified and showed significant improvements in the personal and language domains of the Assessment of Living with Aphasia (ALA, Simmons‐Mackie et al. 2014).

Beales et al. (2016) described generalisation processes after a self‐cueing lexical retrieval intervention across word classes in four participants with PPA (three with svPPA, one with lvPPA). Participants received eight twice‐weekly individual therapy sessions for 90 min over a period of 4 weeks. During word retrieval exercises, autobiographical, semantic, orthographic, and phonological cues were provided by the SLT. Subsequently, an answer template was developed for each item to facilitate independent naming. The QoL‐related changes were assessed using a customised confidence scale. All participants rated their confidence in word retrieval skills and their perception of word retrieval skills higher after the intervention (Beales et al. 2016).

Mahendra and Tadokoro (2020) reported the course of treatment in an individual with nfvPPA over 3 years. The tailored intervention aimed to support communication, social participation, a sense of identity, and emotional well‐being and was aligned with palliative care principles. In the first and second year, therapy took place in intervals (one 12‐week period of therapy per semester and another 6‐week therapy period in summer) containing individual (twice a week) and group therapy (e.g., participating in an aphasia choir for a minimum of once a week). In the third year, one of the two weekly individual sessions took place online, as the participant was no longer able to drive. Therapy approaches ranged from family counselling and education to script training and the use of AAC as a compensatory strategy. In the third year, approaches from life story work (Corsten et al. 2015; Kaiser and Eley 2017) were also used with the aim of improving the participants’ experiences of identity and well‐being. QoL was measured with the ASHA QCLS in Years 1 and 2 and with the AIQ‐21 in Year 3. Improvements of QoL between Years 1 and 2 are described (Mahendra and Tadokoro 2020).

Cadório et al. (2021) presented the results of their structured training in two participants with PPA (one with nfvPPA and one with mixed PPA). Therapy consisted of 12 weekly sessions in two phases: the first phase was based on a restorative approach, and the second phase was based on a compensatory approach. The sessions were held as individual therapy sessions, with care partners present in an observing and supporting role. In the first phase, tasks such as confrontation naming or word‐to‐picture matching were carried out, while in the second phase, role‐playing of everyday situations or training in the use of AAC was performed. QoL was measured by the SAQOL‐39 (Hilari, Byng, et al. 2003), and stable values were found over the course of treatment (Cadório et al. 2021).

Andrade‐Calderón et al. (2015) investigated the effects of speech and language therapy in one participant with nfvPPA. Therapy was conducted for 12 months in weekly sessions and aimed to stimulate phonological, lexical, and syntactic processing. QoL was measured by the DEMQOL (Smith et al. 2007) and the WHOQOL‐BREF (The Whoqol Group 1998), which indicated improvement in QoL and activities of daily living. The results of the Geriatric Depression Scale (GDS, [Yesavage et al. 1982]) indicated improvements in depression. Qualitative feedback from the family indicated an improvement in social and communicative participation (Andrade‐Calderón et al. 2015).

### 3.3 Summary of the Key Findings

All included studies showed at least tendencies towards an improved or stable QoL of the participants over the period of the respective interventions. However, only three out of nine quantitative studies reported significant improvements (Jokel et al. 2017; Whitworth et al. 2018; Rogalski et al. 2016). Comparability of the results is difficult because the approaches varied greatly.

### 3.4 RQ1: QoL‐Enhancing Approaches

The interventions of the included studies mainly aimed at more than one level of the FROM model (Simmons‐Mackie and Kagan 2007). Nine of the selected studies aimed at the activities and participation level (Mahendra and Tadokoro 2020; Kim et al. 2018; Morhardt et al. 2019; Volkmer, Walton, et al. 2023; Jokel et al. 2017; Góral‐Półrola et al. 2015; Cadório et al. 2021; Rogalski et al. 2016; Whitworth et al. 2015), and seven aimed at the body function and body structure level (Mahendra and Tadokoro 2020; Andrade‐Calderón et al. 2015; Beales et al. 2016; Cadório et al. 2021; Jokel et al. 2010; Whitworth et al. 2018; Rogalski et al. 2016). Of these seven studies, three focused exclusively on improving linguistic functions (Andrade‐Calderón et al. 2015; Beales et al. 2016; Jokel et al. 2010), while the remaining four integrated linguistic interventions and were combined approaches (Mahendra and Tadokoro 2020; Cadório et al. 2021; Rogalski et al. 2016). Environmental factors were targeted in five of the included studies and were combined with methods targeting activities and participation levels. Personal factors and identity were addressed in three of the included studies, combined with activity and participation approaches (Mahendra and Tadokoro 2020; Kim et al. 2018; Morhardt et al. 2019). Table 5 shows the allocation of the therapeutic approaches to the different areas of the FROM model.

**TABLE 5:** Allocation of the included studies to the FROM model.

	Kim et al. (2018)	Morhardt et al. (2019)	Jokel et al. (2017)	Góral‐Półrola et al. (2015)	Rogalski et al. (2016)	Volkmer et al. (2023)	Jokel et al. (2010)	Whitworth et al. (2018)	Beales et al. (2016)	Mahendra and Tadokoro (2020)	Cadório et al. (2021)	Andrade‐Calderón (2015)
**Personal factors and identity**	✓	✓								✓		
**Body function and body structure**					✓		✓	✓	✓	✓	✓	✓
**Environment (barriers and facilitators)**			✓	✓	✓	✓				✓		
**Activities and participation**	✓	✓	✓	✓	✓	✓		✓		✓	✓	

According to the defined primary outcomes and intervention aims, QoL was the primary outcome in three of the included studies (Kim et al. 2018; Volkmer, Walton, et al.; Jokel et al. 2017). Furthermore, three studies pursued an explorative approach (Mahendra and Tadokoro 2020; Morhardt et al. 2019; Góral‐Półrola et al. 2015), in which the enhancement of QoL was one of the expected effects of the therapy. Six of the included studies listed the improvement in QoL as a secondary outcome (Andrade‐Calderón et al. 2015; Beales et al. 2016; Cadório et al. 2021; Jokel et al. 2010; Rogalski et al. 2016; Whitworth et al. 2015).

### 3.5 RQ2: QoL Assessments

The ASHA Quality of Communication Life Scale (ASHA QCLS, Paul 2019) was used in three of the included studies (Mahendra and Tadokoro 2020; Jokel et al. 2017, 2010). The Aphasia Impact Questionnaire‐21 (AIQ‐21, Swinburn et al. 2019), the Dementia Quality of Life measure (DEMQOL, Smith et al. 2007), and the Communication Confidence Rating Scale for Aphasia (CCRSA, Babbitt et al. 2011) were used in two studies each. Two studies evaluated the effects on QoL via semistructured interviews (Kim et al. 2018; Rogalski et al. 2016). Some assessments, such as the Stroke and Aphasia Quality of Life Scale‐39 (SAQOL‐39, Hilari, Byng, et al. 2003), the Assessment of Living with Aphasia (ALA, Simmons‐Mackie et al. 2014), the Geriatric Depression Scale (GDS, Yesavage et al. 1982), and the neuropsychiatric inventory (NPI, Cummings et al. 1994), were used in only one of the included studies. Qualitative feedback from participants and observations from therapists were used in four studies (Morhardt et al. 2019; Jokel et al. 2017; Góral‐Półrola et al. 2015; Rogalski et al. 2016). In one study, a self‐administered rating scale relating to domains of QoL was used (Pietersma et al. 2013). Table 6 shows the assessments used in the included studies and the related domains of the overall construct of QoL.

**TABLE 6:** Overview of the assessments measuring QoL used in the included studies.

Assessments	Measured domains	Domains of QoL (Pietersma et al. 2013)	Included studies
*Quantitative self‐assessment questionnaires*			
The ASHA Quality of Communication Life Scale (ASHA QCLS)	Participation in social, vocational or educational activities; communication with friends and family; development of satisfying relationships (Paul 2019)	Self‐esteem; autonomy; good social contacts; being able to perform activities of daily living that are important to oneself	(Mahendra and Tadokoro 2020; Jokel et al. 2017, 2010)
Aphasia Impact Questionnaire‐21 (AIQ‐21)	Communication (speaking, reading, writing); participation; well‐being/emotional state (Swinburn et al. 2019)	Positive emotions	(Mahendra and Tadokoro 2020; Volkmer, Walton, et al. 2023)
Dementia Quality of Life measure (DEMQOL)	Daily activities/looking after yourself; health and well‐being; cognitive functioning; social relationships; and self‐concept (Smith et al. 2007)	Good social contacts; self‐esteem; self‐acceptance	(Volkmer, Walton, et al. 2023; Andrade‐Calderón et al. 2015)
Communication Confidence Rating Scale for Aphasia (CCRSA, Babbitt and Cherney 2010)	Confidence in communication (Babbitt and Cherney 2010)	Self‐esteem; autonomy	(Volkmer, Walton, et al. 2023; Rogalski et al. 2016)
Stroke and Aphasia Quality of Life Scale‐39 (SAQOL‐39)	Physical; psychosocial; communication; energy (Hilari, Byng, et al. 2003)	Mobility; independence; autonomy; being able to perform activities of daily living that are important to oneself; being understood by one's environment	(Cadório et al. 2021)
Assessment of Living with Aphasia (ALA)	Language impairment; participation; personal factors; environmental factors (Simmons‐Mackie et al. 2014)	Being able to perform activities of daily living that are important to you; self‐esteem; enjoying the little things in life	(Whitworth et al. 2018)
WHOQOL‐BREF	Physical health; psychological; social relationships; environment (The Whoqol Group 1998)	Mobility; good social contacts; positive emotions; self‐esteem; being able to perform activities of daily living that are important to oneself	(Andrade‐Calderón et al. 2015)
Bespoke Confidence Scale	For example, stress/frustration level during conversation; confidence in communication (Pietersma et al. 2013)	Positive emotions/being well able to handle negative emotions; self‐esteem; autonomy	(Pietersma et al. 2013)

*Qualitative assessments*			
Semistructured interviews	Activities/participation; identity/feeling; environmental factors (Kim et al. 2018); satisfaction (Rogalski et al. 2016)	Good social contacts; enjoying the little things in life; positive emotions; satisfaction	(Kim et al. 2018; Rogalski et al. 2016)
Qualitative feedback	For example, managing stress and depression; feeling understood (Jokel et al. 2017)	Being well able to handle negative emotions; being understood by one's environment	(Jokel et al. 2017)
Observation/observational field notes	For example, ability to nonverbally communicate/forge contacts; improvement of functioning in everyday life (Góral‐Półrola et al. 2015) Coping with limitations and language decline; dealing with increased dependency; experiencing stigma, expressing resilience, feeling a sense of belonging (Morhardt et al. 2019)	Good social contacts; being understood by one's environment; being well able to handle negative emotions; autonomy	(Morhardt et al. 2019; Góral‐Półrola et al. 2015)

*Note*: The domains of QoL were allocated according to the consensus paper by Pietersma et al. (2013).

Abbreviation: QoL, quality of life.

### 3.6 RQ3: Influencing Factors

Of the included studies, seven took place in individual settings (Góral‐Półrola et al. 2015; Andrade‐Calderón et al. 2015; Beales et al. 2016; Cadório et al. 2021; Jokel et al. 2010; Rogalski et al. 2016; Whitworth et al. 2015), three in group settings (Kim et al. 2018; Morhardt et al. 2019; Jokel et al. 2017), and one in a dyadic constellation (Volkmer, Walton, et al. 2023). One single‐case study described the combined use of individual and group settings (Mahendra and Tadokoro 2020). Three studies described interventions provided by interdisciplinary teams (Kim et al. 2018; Morhardt et al. 2019; Jokel et al. 2017). From the different orientations of the studies, it is possible to identify factors that could be responsible for the therapeutic effects. The factors that influence the therapeutic effects are discussed below.

## 4 Discussion

This study aimed to describe the current state of research on QoL‐enhancing speech and language therapy for people with PPA. Twelve studies were included in the analysis. Most of the included studies were of fair to high quality according to the MMAT. The categorisation of the studies in the evidence hierarchy according to the NHMRC indicates a lack of studies with high‐level of evidence. Two of the included studies were categorised as qualitative, and 10 as quantitative. Although some of the studies used quantitative and qualitative approaches (Jokel et al. 2017; Góral‐Półrola et al. 2015; Rogalski et al. 2016), they were neither labelled as mixed methods nor did they meet the criteria of the MMAT checklist for equal integration of both methods in data collection and analysis. Although Jokel et al. (2017) considered qualitative comments in addition to quantitative results, the method of analysis was not explicitly named. In fact, their research question (the comparison between the intervention group and the control group) cannot be answered by the qualitative results either. The fact that the researchers themselves categorise their study as a ‘comparison group study’ (Swinburn et al. 2019, 53) and report nothing about a mixed methods approach shows that it is more of a quantitative study. Rogalski et al. (2016) and Góral‐Półrola et al. (2015) also combined qualitative and quantitative methods without claiming to use a mixed methods approach. Rather, qualitative and quantitative methods were used independently of each other in these studies. The lack of mixed methods studies in the field of QoL‐enhancing therapy for people with PPA is notable, given the growing importance of this methodology in QoL research for theoretical and methodological reasons such as the multidimensionality and the subjectivity of the construct (Klassen et al. 2012). Kim et al. (2018) also point out that a mixed methods analysis would be suitable to assess the whole impact of Aphasia Camp participation. The following sections present a discussion of QoL‐enhancing approaches, the QoL assessments and the influencing factors of the included studies. This is to provide answers to the research questions previously posed.

### 4.1 RQ1: QoL‐Enhancing Approaches

In all included studies, QoL was measured as an outcome, although it was not the primary treatment goal in all studies. The variety of approaches is reflected in the allocation of approaches to levels in the FROM model (see Figure 2). In contrast to the evidence included by Wauters et al. (2023), the majority of the studies in our scoping review aimed to achieve an improvement at the level of activity and participation (*n* = 9). In contrast, only three studies were categorised as exclusively aiming at body function and body structure (Andrade‐Calderón et al. 2015; Beales et al. 2016; Jokel et al. 2010). This finding suggests that the identified studies were more focused on improvements in everyday functions compared to the range of other therapies offered for PPA. Only the study by Mahendra and Tadokoro (2020), which took a palliative approach, covered all levels of the FROM model (Mahendra and Tadokoro 2020). This might be because an entire course of therapy in a person with nfvPPA over 3 years is reported, which requires an adaptation of the therapeutic methods over time (Mahendra and Tadokoro 2020). However, it is controversial whether a focus on QoL always implies a palliative perspective and whether identity work approaches should only be applied at late stages of PPA. Although the QoL‐perspective might become more important in later stages of the disease, according to best practice principles for people with PPA (Volkmer, Cartwright, et al. 2023), problems such as the manifestation of depression should be prevented by early psychosocial intervention.

It is not possible to state which intervention was most suitable for improving QoL due to the methodological differences. Based on the idea that the presence of a language disorder has a negative impact on an individual's identity and self‐image (Shadden 2005), this level might be essential for improving QoL. This argument is in line with Davies et al. (2024), who promote the adaptation of interventions to support identity renegotiation for people with PPA based on their qualitative data (Davies et al. 2024). Work is currently underway to evaluate the efficacy of a biographic‐narrative approach tailored to the needs of people with PPA, with the aim of improving QoL (‘Cope PPA,’ Gauch et al. 2024). However, the allocation of the therapeutic approaches according to the FROM model shows that studies targeting personal factors and identity are under‐represented in our sample (see Table 5). According to the model of Doogan et al. (2018), the effects of aphasia and its associated cognitive impairment are as important as the social isolation and low mood that emerge from it. These factors reduce the QoL of people with aphasia. The model illustrates that an intervention to improve QoL should go beyond impairment‐based approaches (Doogan et al. 2018). The FROM model can be used to demonstrate that an improvement in QoL requires a holistic approach because otherwise, not all constituent elements are addressed.

Findings further indicate that therapy targeting the level of body function and structure might have positive effects on the perception of effectiveness, which covers part of the construct of QoL. For example, all participants in the study by Beales et al. (2016) achieved better scores for communication confidence after a self‐cueing intervention (Beales et al. 2016). A high level of participation in social life by people with PPA was seen as a predictor of better QoL in some of the included studies (Andrade‐Calderón et al. 2015). However, the results of the included studies once again show that not all people experience improved QoL as a result of improved communication. Even if previously formulated therapy goals are achieved, this does not necessarily result in an improved QoL (Volkmer, Walton, et al. 2023). This finding is consistent with studies on people with post‐stroke aphasia (Franzén‐Dahlin et al. 2010) and emphasises the importance of interventions aiming specifically at improving QoL (Doogan et al. 2018).

### 4.2 RQ2: QoL Assessments

QoL in the included studies was measured with a variety of assessments. While some conventional QoL assessments, such as the ASHA‐QCLS, DEMQOL, SAQOL‐39, ALA and WHOQOL‐BREF, were used, other scales have been used that cover only single domains of QoL, such as the CCRSA. Whether an assessment covers only one or several domains of the QoL should be taken into account when considering the suitability of that assessment. The question of whether an assessment is appropriate for assessing QoL is still difficult to answer and depends on the respective study aim and the assumed underlying mechanism of therapy.

In qualitative evaluation, whether or not QoL domains were covered depends on the interviewing guidelines or observational field note guides used. If questions cover QoL‐related domains, an interview might provide deeper insights into a personal perspective and enable personal relevance to be established. The use of qualitative methods therefore has the potential to capture aspects of QoL that are not covered by quantitative measurement instruments (Abbey et al. 2011).

The use of proxy‐ratings and observational methods in conjunction with the assessment of QoL should be given careful consideration. As QoL is always subjective, external assessors would need high levels of empathy to take the perspective of the person with PPA. Ruggero et al. (2023) point out that self‐ratings and proxy‐ratings of QoL in PPA patients are not interchangeable, although exact patterns need to be investigated in future studies (Ruggero et al. 2023). Furthermore, there is evidence indicating that people with dementia report their QoL to be significantly better than what observational measures suggest (Edelman et al. 2005). Against the background of advanced PPA with existing mutism, however, the procedure of proxy‐rating seems entirely plausible, so the study by Góral‐Półrola et al. (2015) was included in the present review (Góral‐Półrola et al. 2015). Furthermore, it cannot be denied that the additional external perception of a close person can be helpful in the assessment of negative emotions, especially if there is a communication impairment.

The fundamental question arises as to how QoL can be measured in the case of severe communication impairments. Kaiser and Eley (2017) recommend the use of visual approaches such as the photovoice method or video ethnography, which are nonverbal methods used to provide valuable insights into the lived experiences of people and their social environment (Annang et al. 2016; Wang and Burris 1997; Collier et al. 2016).

### 4.3 RQ3: Influencing Factors

The influence of various factors on the outcomes of the included studies can be discussed. There are indications that (1) personality traits and (2) symptoms/comorbidities of participants; (3) (soft) skills of therapists and their attitudes; (4) therapy methods and materials; (5) treatment doses and (6) setting of therapy may have impacted the improvement in QoL.

The *personality traits of participants* (e.g., resilience, proactivity and positivity) are thought to influence the impact of the intervention on their QoL (Kim et al. 2018; Cadório et al. 2021). Most likely, the personal characteristics of the person with PPA in the study by Kim et al. (2018) helped her to expand her social connections and to take up new activities, which in turn led to an increased QoL (Kim et al. 2018). The fact that an individual still has his or her own goals (e.g., ‘wanting to leave behind a chronicle of her life’ [Franzén‐Dahlin et al. 2010, 21]) might indicate optimism and an existing future perspective, which is possibly related to greater satisfaction (Lennings 2000). Evidence on the future prospects of older people supports this connection and emphasises the importance of achievable therapy goals (Cress and King 1999).

In addition to personal characteristics, it could be assumed that the *symptoms and comorbidities* of PPA influence the therapeutic effect. In fact, this association appears to be highly individualised. Cadório et al. (2021) described how a participant with a prior history of depression maintained good health‐related QoL, which was attributed by the authors to family support and speech and language therapy (Cadório et al. 2021). Previous studies in people with Alzheimer's disease have also shown that the presence of depression does not prevent therapy‐induced improvements in QoL, which can be achieved through reminiscence therapy (Yale 1999). In the study by Rogalski et al. (2016), the severity of PPA was a barrier to web‐based therapy for one participant and prevented participation in their QoL‐enhancing intervention (Rogalski et al. 2016). In contrast, Mahendra and Tadokoro (2020) described how telepractice, even at an advanced stage, helped to enable participation (Mahendra and Tadokoro 2020).

Some studies indicate that the *(soft) skills of therapists and their attitudes* are also decisive for the success of therapy and possibly also for the improvement of QoL. Due to the inclusion criteria, SLTs conducted the interventions in all 12 studies. Additionally, some studies have described the benefits of an interdisciplinary team consisting of social workers, cognitive neuroscientists, psychologists, physiotherapists, occupational therapists, and so forth (Kim et al. 2018; Morhardt et al. 2019; Jokel et al. 2017). Beales et al. (2016) recommend careful consideration of the course of the disease when making clinical decisions (Beales et al. 2016). Mahendra and Tadokoro (2020) argue for the consideration of palliative principles in the management of people with PPA, such as arranging continuity of care when ‘“curative” therapies are no longer useful’ (Franzén‐Dahlin et al. 2010, 9). These points require a certain flexibility from therapists in treatment planning (Andrade‐Calderón et al. 2015) and fit into the principles and philosophies of Volkmer, Cartwright, et al. (2023), who proposed aspects such as the importance of ‘knowing people deeply,’ ‘preventing disasters,’ being practical and connected across disciplines (Kim et al. 2018, 1074).

Some of the discussed studies mentioned *therapy materials* as influencing factors. The authors refer to different materials, such as pictures and photographs of loved ones (Morhardt et al. 2019; Rogalski et al. 2016) or digital aids (Kim et al. 2018; Góral‐Półrola et al. 2015). Self‐designed communication books are described as helpful (Rogalski et al. 2016), which is in line with existing evidence that favours the early introduction of self‐developed tools (Cress and King 1999). In the study by Kim et al. (2018), the participants benefited from the strategies of Supported Conversation for Adults with Aphasia (SCA, Aphasia Institute 2025). The set of techniques offered there was described by the couple as a helpful ‘toolbox’ (Kim et al. 2018, 275). In the study by Morhardt et al. (2019), participants favoured practical sessions over the more theoretical sessions, from which the authors conclude that talk‐based therapy for people with PPA has its limitations (Morhardt et al. 2019).

The interventions described in our scoping review had different *treatment doses*. The studies aiming to improve the level of body function and structure were carried out at a greater frequency than those aimed at personal factors or activities and participation. This difference becomes particularly apparent when comparing the Aphasia Camp (Kim et al. 2018) with the errorless learning approach according to Jokel et al. (2010). There is a trend towards less frequent interventions in people with PPA compared to the best practices for post‐stroke aphasia, where high‐frequency interventions of ≥ 10 h per week are recommended (Breitenstein et al. 2017; Brady et al. 2022). The results of this scoping review are therefore consistent with those of a recently published systematic review about behavioural interventions in people with PPA with heterogeneous frequencies of therapy, approximately 2–6 times per week (Wauters et al. 2023), and a systematic review that suggests weekly sessions at 5–12 week intervals for the treatment of people with dementia (Hockley et al. 2023). In contrast to these findings, some experts from the field of aphasiology suggest that interventions aiming at the level of personal factors and identity require time to show positive effects (Corsten et al. 2015). The goals of the individual patient sample should always be weighed when dosing procedures are performed (Abbey et al. 2011).

The *setting of the therapies* varied. Due to the heterogeneous assessments and statistical analysis of the included studies, it is not possible to determine whether group therapy, individual therapy or dyadic interventions are better suited to improving QoL in people with PPA. However, according to our results, it seems likely that all three settings have the potential to improve QoL. The number of people receiving group therapies varied between five and nine participants with PPA per therapy group (Morhardt et al. 2019; Jokel et al. 2017). To our knowledge, there is no evidence‐based recommendation on the appropriate group size for group therapy with people with PPA, and other reviews show a wide range of participant numbers (Watanabe et al. 2024). Regarding the inclusion of carers, the results are heterogenous. On the one hand, participants in the study by Morhardt et al. (2019) stated that they wanted to involve their care partners more (Morhardt et al. 2019), and Rogalski et al. (2022) describe care partner involvement as a relevant factor that influenced the efficacy of their intervention, supporting the use of dyadic interventions (Rogalski et al. 2016, 2022). On the other hand, separation into groups of individuals with PPA and their partners was described as a positive experience in the study by Jokel et al. (2017).

### 4.4 Limitations

While this study offers valuable insights, there are several aspects that future research could address. One limitation is the search strategy, which utilised ‘primary progressive aphasia’ as a search term. This may have excluded studies using alternate terms like ‘semantic dementia’ or ‘progressive aphasia.’ The search strategy by Ruggero et al. (2019) could serve as a positive example in this context (Ruggero et al. 2019). As the search strategy was developed to obtain an overview of the current study landscape, it might not fully capture the complexity of the task of covering all constructs of QoL. In the future, a systematic review could be written on this topic, in which the 25 domains of QoL are also included in the initial research in a systematic way. In such a systematic review, the influencing factors, like therapy settings or the frequency of therapy, could be addressed in a structured manner. There was no PROSPERO registration of the review protocol, which is not mandatory for scoping reviews (Munn et al. 2018). Nevertheless, we would recommend pre‐registration for a future review in this area and developing the search string in collaboration with a librarian (Peters et al. 2020).

Another limitation in the search strategy was that, due to a lack of resources, only the database search was carried out by two researchers, while the first author conducted the grey literature search alone, which also entails a risk of bias. The fact that different QoL assessments were used in the studies posed challenges for result comparison. Nonetheless, incorporating both quantitative and qualitative approaches in the current scoping review seems appropriate for obtaining a comprehensive overview.

Two abstracts were excluded because of a missing full text at the time of analysis. We further referred exclusively to studies published in English‐language journals. For this reason, and because of the search strategy, we have not considered findings from the doctoral theses of Jade Cartwright (2013) and Jacqueline Kindell (2015). Consequently, there is a risk of bias in our analysis due to the omitted evidence.

## 5 Conclusions

The present scoping review identified relevant literature referring to the potential of speech and language therapy to improve QoL in people with PPA. The results were analysed on a content and methodological level. There is a clear need for high‐quality controlled trials of QoL‐enhancing interventions, best with a mixed methods approach. The evaluation of the interventions and categorisation using the FROM model shows a lack of studies that specifically focus on the identity of people with PPA or interventions which directly target QoL. It is possible that a focus on this domain could lead to the person‐centred approach experts are already calling for (Volkmer, Cartwright, et al. 2023). The fact that there are numerous individual case studies in research for people with PPA certainly has to do with the rarity of the condition, but it could also be due to the fact that person‐centred therapy that addresses the needs of the individual is rarely implemented in large studies. The question arises as to whether studies with larger samples might also allow for individualised therapy focusing on domains such as identity and taking into account personal circumstances, mechanisms and meaningful outcomes (Rogalski et al. 2022). Such an approach could have a greater impact on QoL considering that the domains of self‐acceptance, self‐esteem, and good social contact are strongly linked to the concept of identity and, consequently, to QoL. The authors of the present review agree with Pietersma et al. (2013), who call for increased attention to social and mental domains (Pietersma et al. 2013). The present review shows that there is a lack of standardised QoL assessments for people with PPA. In addition to the influence of the respective method, further influencing factors (e.g., treatment doses or setting of therapy) were identified and discussed. The results of the present scoping review prompt the planning of future empirical studies and reviews in which a more differentiated view of the relationships between QoL improvements and the type of intervention should be taken.

## Ethics Statement

None for this paper, as it is a scoping review of primary research evidence.

## Consent

The authors have nothing to report.

## Conflicts of Interest

The authors declare no conflicts of interest.

## Supporting information




**Supporting File**: Supp‐Info‐sup‐0001‐SuppMat.docx

## Data Availability

The full data extracted and evaluated are available from the authors upon reasonable request.

## Endnotes

1 The Reliable Change Index (RCI) was introduced by Jacobson et al. (1984) and is defined as the expected amount of change on a given scale, which exceeds the attributed measurement error (Blampied 2022).

2 The first author of the article has been contacted to obtain this information.
